# Supplementary material for: Determinants of Household-Level Food Storage Practices and Outcomes on Food Safety and Security in Accra, Ghana
Source: Foods. 2022 Oct 19;11(20):3266. doi: 10.3390/foods11203266 (PMC9601939; doi:10.3390/foods11203266)
Supplement: Supplementary file 1 [file foods-11-03266-s001.zip › foods-1955928-supplementary.pdf]

Table S1. Measurement model reliability and validity tests.

Variables	Composite Reliability	Average Variance Extracted (AVE)
1. Feeding and Cooking Behavior	1.00	1.00
2. Food Choice Motives	0.91	0.83
3. Food Handling	0.71	0.58
4. Food Infrastructure	0.71	0.56
5. Food Safety and Health	0.78	0.65
6. Food Security	0.81	0.59
7. Food Shopping Behavior	0.75	0.60
8. Food Storage	0.88	0.59
9. Food Storage Knowledge	0.85	0.73
10. Food Waste Management	0.80	0.66
11. Household Head Income	1.00	1.00
12. Household Size	1.00	1.00
13. Household Socio-economic Status	1.00	1.00
14. Income on Food	0.98	0.91

Table S2. PLS cross loadings.

	Feeding and Cooking Behavior	Food Choice and Motives	Food Handling	Food Infrastructure	Food Safety and Health	Food Security	Food Shopping Behavior	Food Storage	Food Storage Knowledge	Food Waste Management	House–Hold Head Income	House–Hold Size	House–Hold Socio–Economic Status	Income on Food
FCOOK_1	1.00	0.43	−0.32	0.14	−0.24	0.36	−0.26	−0.38	−0.36	−0.31	−0.43	−0.19	−0.49	0.35
FCHOICE_1	0.35	0.91	−0.46	0.32	−0.33	0.58	−0.28	−0.64	−0.58	−0.43	−0.82	−0.18	−0.79	0.55
FCHOIC_2	0.42	0.91	−0.47	0.33	−0.41	0.63	−0.35	−0.67	−0.51	−0.52	−0.73	−0.27	−0.84	0.60
FHANDL_1	−0.17	−0.08	0.84	−0.12	0.22	−0.17	0.20	0.25	0.17	0.12	0.10	−0.01	0.09	−0.14
FHANDL_2	−0.30	−0.54	0.96	−0.55	0.52	−0.43	0.50	0.73	0.64	0.46	0.54	0.25	0.54	−0.36
INFRAST_1	0.18	0.27	−0.25	0.54	−0.12	0.31	−0.11	−0.27	−0.25	−0.20	−0.35	−0.09	−0.29	0.26
INFRAST_2	0.08	0.29	−0.50	0.91	−0.35	0.21	−0.46	−0.50	−0.60	−0.35	−0.35	−0.42	−0.32	0.12
INFRAST_3	0.18	0.27	−0.25	0.54	−0.12	0.31	−0.11	−0.27	−0.25	−0.20	−0.35	−0.09	−0.29	0.26
INFRAST_7	0.08	0.29	−0.50	0.91	−0.35	0.21	−0.46	−0.50	−0.60	−0.35	−0.35	−0.42	−0.32	0.12
FSAFE_1	−0.14	−0.13	0.37	−0.18	0.70	−0.11	0.40	0.30	0.32	0.37	0.13	0.17	0.17	−0.08
FSAFE_2	−0.24	−0.47	0.47	−0.35	0.89	−0.40	0.34	0.48	0.46	0.43	0.46	0.20	0.46	−0.35
FSECUR_1	0.35	0.64	−0.48	0.46	−0.40	0.74	−0.33	−0.66	−0.56	−0.38	−0.67	−0.19	−0.64	0.53
FSECUR_2	0.30	0.56	−0.32	0.17	−0.26	0.95	−0.20	−0.48	−0.34	−0.35	−0.60	0.14	−0.60	0.95
FSECUR_3	0.30	0.56	−0.32	0.17	−0.26	0.95	−0.20	−0.48	−0.34	−0.35	−0.61	0.13	−0.60	0.95
FSHOP_1	−0.18	−0.18	0.19	−0.23	0.31	−0.15	0.63	0.28	0.30	0.24	0.16	0.26	0.23	−0.15
FSHOP_2	−0.23	−0.33	0.52	−0.41	0.38	−0.26	0.90	0.51	0.50	0.30	0.34	0.21	0.32	−0.21
FSTORE_1	−0.26	−0.58	0.55	−0.60	0.44	−0.50	0.42	0.74	0.66	0.47	0.65	0.31	0.58	−0.42
FSTORE_2	−0.26	−0.40	0.50	−0.38	0.34	−0.29	0.32	0.65	0.43	0.34	0.37	0.21	0.41	−0.26
FSTORE_3	−0.31	−0.61	0.58	−0.32	0.32	−0.52	0.38	0.81	0.55	0.36	0.59	0.15	0.62	−0.48
FSTORE_4	−0.40	−0.64	0.67	−0.45	0.49	−0.54	0.50	0.88	0.68	0.47	0.64	0.28	0.67	−0.51
FSTORE_5	−0.20	−0.51	0.47	−0.30	0.32	−0.49	0.39	0.75	0.52	0.37	0.47	0.22	0.55	−0.45
FSKNOW_1	−0.29	−0.38	0.46	−0.59	0.42	−0.33	0.46	0.51	0.81	0.37	0.44	0.36	0.40	−0.23
FSKNOW_2	−0.33	−0.61	0.59	−0.48	0.44	−0.47	0.46	0.74	0.90	0.42	0.66	0.33	0.64	−0.43
FWASTE_1	−0.26	−0.46	0.38	−0.37	0.44	−0.37	0.33	0.48	0.45	0.87	0.42	0.28	0.52	−0.35
FWASTE_2	−0.24	−0.38	0.35	−0.23	0.37	−0.30	0.22	0.37	0.29	0.76	0.36	0.11	0.39	−0.28
INCOME_HH	−0.43	−0.85	0.51	−0.44	0.41	−0.71	0.34	0.72	0.66	0.48	1.00	0.23	0.86	−0.69
HH_SIZE	−0.19	−0.25	0.22	−0.39	0.23	0.02	0.28	0.31	0.39	0.26	0.23	1.00	0.34	0.10
STATUS_HH	−0.49	−0.90	0.51	−0.40	0.42	−0.70	0.36	0.74	0.63	0.56	0.86	0.34	1.00	−0.69
FINCOM_2	0.37	0.64	−0.36	0.22	−0.31	0.83	−0.24	−0.58	−0.41	−0.39	−0.71	0.06	−0.71	0.96
FINCOM_3	0.30	0.56	−0.32	0.17	−0.26	0.95	−0.20	−0.48	−0.34	−0.35	−0.61	0.13	−0.60	0.95
FINCOM_4	0.30	0.56	−0.32	0.17	−0.26	0.95	−0.20	−0.48	−0.34	−0.35	−0.60	0.14	−0.60	0.95
FINCOM_5	0.37	0.65	−0.36	0.23	−0.31	0.83	−0.24	−0.58	−0.41	−0.40	−0.71	0.05	−0.72	0.96

Note: Shaded cells indicate cross-loadings of items on own constructs.

Table S3. Network analysis and path coefficients of determinants of household-level food storage practices.

Variables	Path Coefficient	*p* Values	Remark
Food Storage Knowledge -> Food Storage	0.268	0.000	Supported
Cooking Behavior -> Food Storage	−0.005	0.851	Not Supported
Food Choice Motive -> Food Storage	−0.356	0.000	Supported
Food Shopping Behavior -> Food Storage	0.088	0.006	Supported
Food Storage Knowledge -> Food Handling	0.625	0.000	Supported
Food Handling -> Food Storage	0.308	0.000	Supported
Food Choice Motive -> Food Infrastructure	0.240	0.000	Supported
Food Shopping -> Food Infrastructure	−0.349	0.000	Not supported
Food Infrastructure -> Food Storage	−0.045	0.162	Not Supported
Food Storage -> Food Waste Management	0.527	0.000	Supported
Food Storage -> Food Safety	0.504	0.000	Supported
Food Storage -> Expenditure on Food	−0.559	0.000	Not Supported
Food Storage -> Perceived Food Security	−0.657	0.000	Not Supported
